# Strain Gated Bilayer Molybdenum Disulfide Field Effect Transistor with Edge Contacts

**DOI:** 10.1038/srep41593

**Published:** 2017-02-10

**Authors:** Yu Chai, Shanshan Su, Dong Yan, Mihrimah Ozkan, Roger Lake, Cengiz S. Ozkan

**Affiliations:** 1 Materials Science and Engineering Program, University of California, Riverside, CA 92521 USA; 2 Department of Electrical and Comp. Engineering, University of California, Riverside, CA 92521 USA; 3 Center for Nanoscale Science & Engineering, University of California, Riverside, CA 92521 USA; 4 Department of Mechanical Engineering, University of California, Riverside, CA 92521 USA

## Abstract

Silicon nitride stress capping layer is an industry proven technique for increasing electron mobility and drive currents in n-channel silicon MOSFETs. Herein, the strain induced by silicon nitride is firstly characterized through the changes in photoluminescence and Raman spectra of a bare bilayer MoS_2_ (Molybdenum disulfide). To make an analogy of the strain-gated silicon MOSFET, strain is exerted to a bilayer MoS_2_ field effect transistor (FET) through deposition of a silicon nitride stress liner that warps both the gate and the source-drain area. Helium plasma etched MoS_2_ layers for edge contacts. Current on/off ratio and other performance metrics are measured and compared as the FETs evolve from back-gated, to top-gated and finally, to strain-gated configurations. While the indirect band gap of bilayer MoS_2_ at 0% strain is 1.25 eV, the band gap decreases as the tensile strain increases on an average of ~100 meV per 1% tensile strain, and the decrease in band gap is mainly due to lowering the conduction band at K point. Comparing top- and strain-gated structures, we find a 58% increase in electron mobility and 46% increase in on-current magnitude, signalling a benign effect of tensile strain on the carrier transport properties of MoS_2_.

Strain is a critical ingredient in modern transistor scaling. For Intel process technologies, the electron mobility decreased from 400 to 120 cm^2^/Vs when the industry migrated from 0.80 μm to 0.13 μm technology node due to the large vertical electric field^1^. Strain engineering has proven an effective route for mobility enhancement by modifying the carrier effective mass and mean scattering time^2^. For transistors with sub-100 nm gate length, strained silicon increased the saturated MOSFETs drive currents by 10–20% and mobility by >50%, and later ramped into high volume manufacturing on high performance microprocessors in the 90 nm logic technology^3^. The electron and hole mobility of silicon responds differently to externally applied stress. Longitudinal tensile stress along transistor channel improves electron mobility but degrades hole mobility^4^,^5^,^6^. p-MOSFET features a compressively strained SiGe film embedded in the source and drain regions^7^. The mismatch in the SiGe to Si lattice causes the channel to be under a uniaxial compressive strain, leading to significantly improved hole mobility^8^. For n-MOSFET, a post-salicide tensile silicon nitride capping layer was deposited on top of the transistor gate, wrapping both the gate and source drain area^7^. As a tensile stressor inside the nitride film tends to shrink, the stressor on the source and drain pulls apart the ends of the transistor channel and mainly produce a longitudinal tensile strain in the n-MOSFET channel^2^.

For three-dimensional semiconductor, the ultimate strain exertion is limited by both bulk defects and surface imperfections^9^. Transition metal dichalcogenides (TMDCs) are more suitable for strain engineering for two reasons: negligible bulk defects because of a thickness of only a few atomic planes and a fully-terminated surface, eliminating fab processing steps that usually targeted at the passivation of dangling bonds. A breaking strain up to 11% for MoS_2_^10^ has been proved experimentally, whereas bulk silicon can be strained only 1.2% before fracture^9^.

Theoretical studies have predicted that when an external tensile stress is applied, the electronic structure of monolayer MoS_2_ undergoes a series of variations: first, a direct-to-indirect band gap transition when the lattice constant is just slightly lengthened; second, a more drastic semiconductor-to-metal transition when the lattice constant is increased by more than 9.8%^11^. In particular, the tensile strain reduces the gap energy and the effective masses while the compressive strain enhances them^11^,^12^. Majority of experimental demonstration of the strain effect on MoS_2_ employs standard three-point or four-point bending apparatus together with micro-Raman facilities. Ultra-thin MoS_2_ samples are firstly exfoliated and then clamped onto a bendable material such as polydimethylsiloxane (PDMS)^13^, SU8/polycarbonate^14^, polyethylene terephthalate (PET)^15^ and poly (methyl methacrylate) (PMMA)^16^. Photoluminescence (PL) spectra are recorded at the moment when a mechanical strain is exerted. Similar findings from the aforementioned literature have been reported: a red shift of PL emission energy and decreased peak intensity under uniaxial tensile strain, conforming to the direct-to-indirect transition of the optical band gap as predicated by theoretical studies. The observation can be qualitatively understood as a result of reduced orbital overlap and hybridization due to weakened atomic bonds^16^. In complementary to the above results, a blue shift of the PL peak and an increase of the emission intensity have been reported for biaxial compressive strain exerted to tri-layer MoS_2_ through a piezoelectric substrate^17^. At the device level, back-gated MoS_2_ transistors on a flexible substrate measured in the stretched state shows a shift of the transfer curve toward lower back-gate voltages and an increase in electron current than the results measured in flat state^18^.

In this paper, we explore on the concept of “strain-gated” MoS_2_ MOSFET. Here, strain is exerted to MoS_2_ channel through the deposition of a silicon nitride stress capping layer that covers the entire transistor active area, analogous to the industry-proven technique applied to the early generation of n-channel silicon transistors. To enhance the field-effect mobility, uniaxial tensile strain along the transistor channel is favored to be generated in order to reduce the band gap and electron effective mass. Current on/off ratio and other performance metrics are measured as the transistors evolve from back-gated, to top-gated and finally, strain-gated structure.

## Results and Discussion

**Figure 1:**
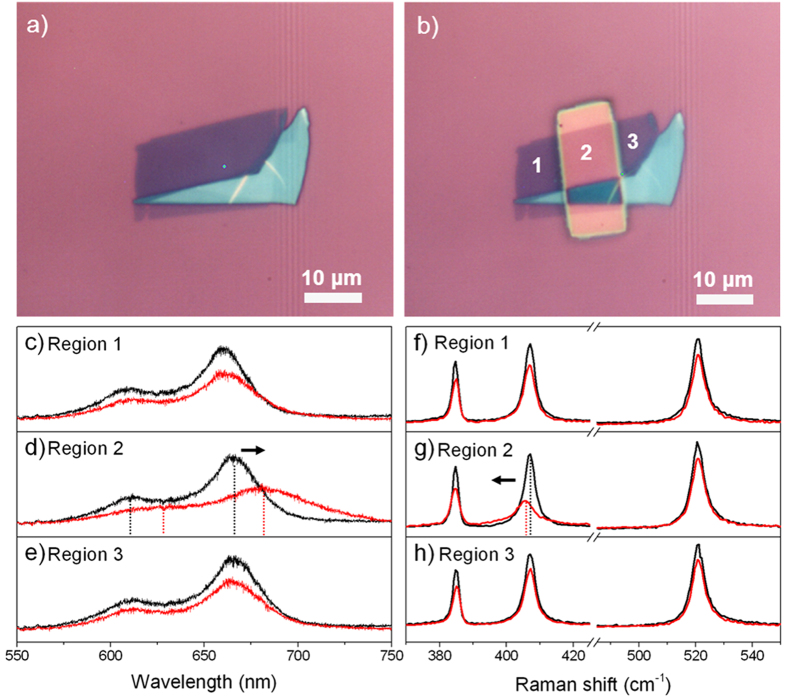
Photoluminescence and Raman spectra of bi-layer MoS_2_ with locally deposited silicon nitride. (**a**) the as-prepared bi-layer MoS_2_ on Si/SiO_2_ substrate; (**b**) the same sample with only region 2 covered by silicon nitride, region 1 and 3 serve as control groups; (**c** to **e**) photoluminescence and (**f** to **h**) Raman spectra of all three regions, spectra in black color were taken from the as-prepared sample; red color, post-nitride deposition process. The black arrows indicate the direction of peak shift.

First, we design sample structure to visualize the internal stress of the silicon nitride indirectly through the Raman mode shift of MoS_2_ underneath. Silicon nitride (SiN_x_) deposited by plasma-enhanced chemical vapor deposition (PECVD) are used in both Raman measurement and transistor characterization. As the ions do not respond to the field at a single RF frequency of 13.56 MHz, a thin film deposited under such conditions typically exhibits tensile stress^19^,^20^. As shown in Fig. 1(a) and (b), a bi-layer MoS_2_ sample was exfoliated and transferred onto a Si/SiO_2_ substrate. The sample is divided into three regions: only region 2 is covered by 125 nm PECVD silicon nitride; the intact regions 1 and 3 serve as control groups. This is to minimize potential non-uniformities from different pristine bi-layer samples.

Before nitride deposition, the bi-layer thickness and the homogeneity of the MoS_2_ sample are confirmed by PL and Raman measurement. In all three regions, PL spectra show the two prominent emission peaks at 670 and 627 nm (Fig. 1c–e), corresponding to the two resonances known as A1 and B1 excitons^21^, and the Raman spectra show a wavenumber difference of 22 cm^−1^ between the 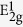 and 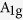 peak (Fig. 1f–h), a signature of bi-layer MoS_2_^22^. To show the similarities in peak intensity among the three regions, the spectra taken from the as-prepared sample in Fig. 1 are re-plotted in overlapping format in Fig. S1 (Supplementary Information).

**Figure 2:**
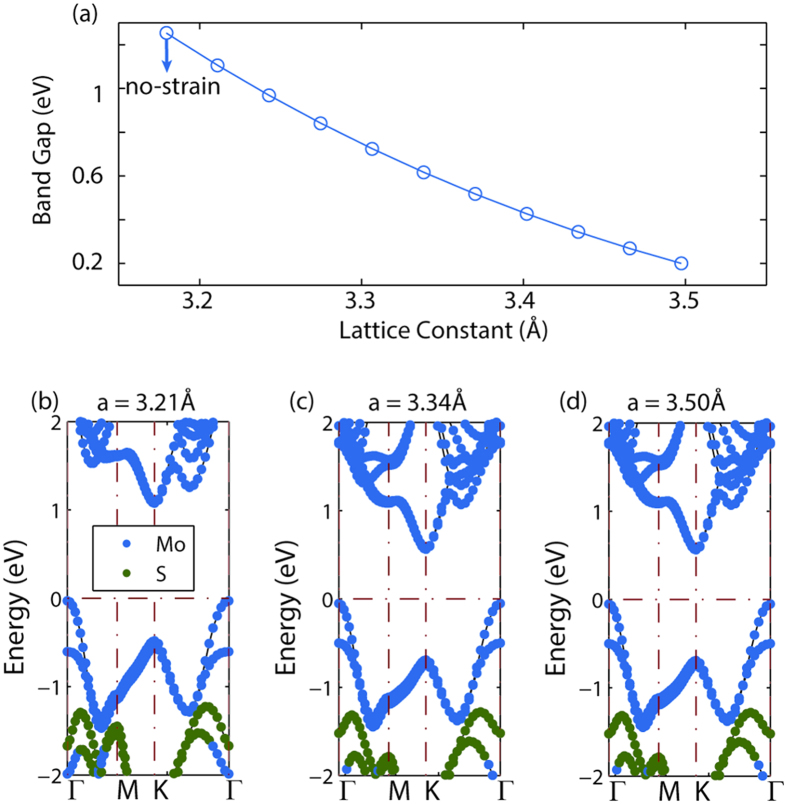
Strained bi-layer MoS_2_ band structure calculation. (**a**) The continuously narrowed band gap of bilayer MoS_2_ as a function of increasing lattice constant, the band structure with 1%, 5% and 10% tensile strain are shown in (**b**,**c** and **d**). The blue and green dots in the figure represent Mo and S element occupation respectively.

In the post-deposition PL spectrum of region 2 (Fig. 1d), we find ~10 nm red shift of the A1 excitons (from 670 to ~680 nm), and about 40% decrease of the emission intensity. The decrease in peak emission energy is ~27 meV by using E = 1240/λ. The findings indicate a narrowed indirect bandgap possibly due to the tensile stress from the nitride capping layer. Difference in thermal expansion coefficient between MoS_2_ and silicon nitride could be another source of stress^23^. As the nitride layer was deposited at 120 °C, a tensile strain can be induced in the MoS_2_ flake during the subsequent cooling of the sample to room temperature. For the Raman spectra, given the fact that the referential peak of silicon remains at 520.7 cm^−1^ before and after nitride deposition, the post-deposition spectrum of region 2 shows a red shift of the 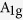 peak (Δω ≈ 1.5 cm^−1^), while the shift of 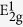 peak is negligible (Fig. 1g). We also observed this shift of A_1g_ peak in other locations on the MoS_2_ sample covered by the nitride slab, however no spatial correlations are concluded. The shift of the A_1g_ peak also disappears once the laser crosses either left or right boundary of the nitride slab. This change in Raman spectra could be due to an extra strain in the out-of-plane direction^13^, but we are still trying to identify the reason behind. Regarding Region 1 and 3, no noticeable peak shift was observed either in PL or Raman characterization, the slightly dropped intensity might originate from some resist reside adsorbed on the MoS_2_ surface at the lift-off step. The continuously narrowed band gap of bi-layer MoS_2_ under tensile strain is calculated and shown in Fig. 2(a). The indirect band gap of bi-layer MoS_2_ at 0% strain is 1.25 eV. It is clear that the band gap decreases as the tensile strain increases, the tunability on average is ~100 meV per 1% tensile strain. Beyond 10%, the bi-layer MoS_2_ finally becomes close to metallic. Based on the simulated result, the magnitude of the tensile strain induced by the nitride capping layer is estimated to be much less than 1%. From the study of band structure in Fig. 2(b–d), the decrease in band gap is mainly coming from the lowering of the conduction band at K point. The band structure keeps indirect through all different tensile strain conditions. And the contribution of the band edge comes purely from Mo element’s d-orbital.

**Figure 3:**
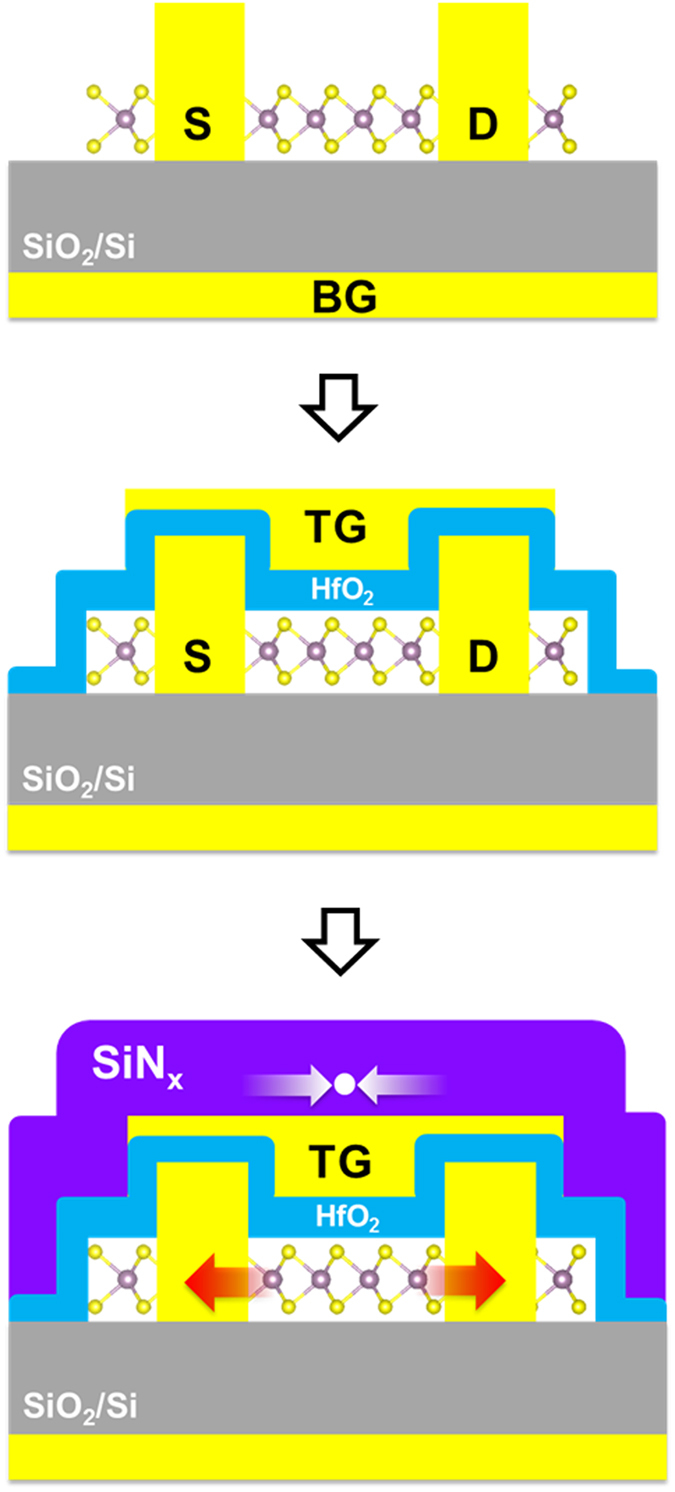
The three main stages for electrical characterization: (**a**) back-gate; (**b**) top-gate and (**c**) strain-gate. As a tensile stressor inside the nitride film tends to shrink, the stressor on the source and drain pulls apart the ends of the transistor channel and mainly produce a longitudinal tensile strain in the MOSFET channel^2^. The sites for source drain contacts are engraved on the bi-layer MoS_2_.

Next, we design experiments to apply the nitride stress capping layer onto MoS_2_ field-effect transistors and observe the strain’s influence on device performance. Figure 3 shows the three main stages for electrical characterization, denoted as back-gate (BG), top-gate (TG) and strain-gate (SG). The key challenge for achieving the final strain-gate configuration is fabricating MoS_2_ FETs with robust electrical contacts to endure multiple fab processing and electrical measurement cycles. From our experience, the yield of working FET and the performance repeatability are significantly improved with one-dimensional edge contacts^24^, in contrast to conventional surface contacts which are affected by inherent surface defects found on natural MoS_2_ crystal^25^.

**Figure 4:**
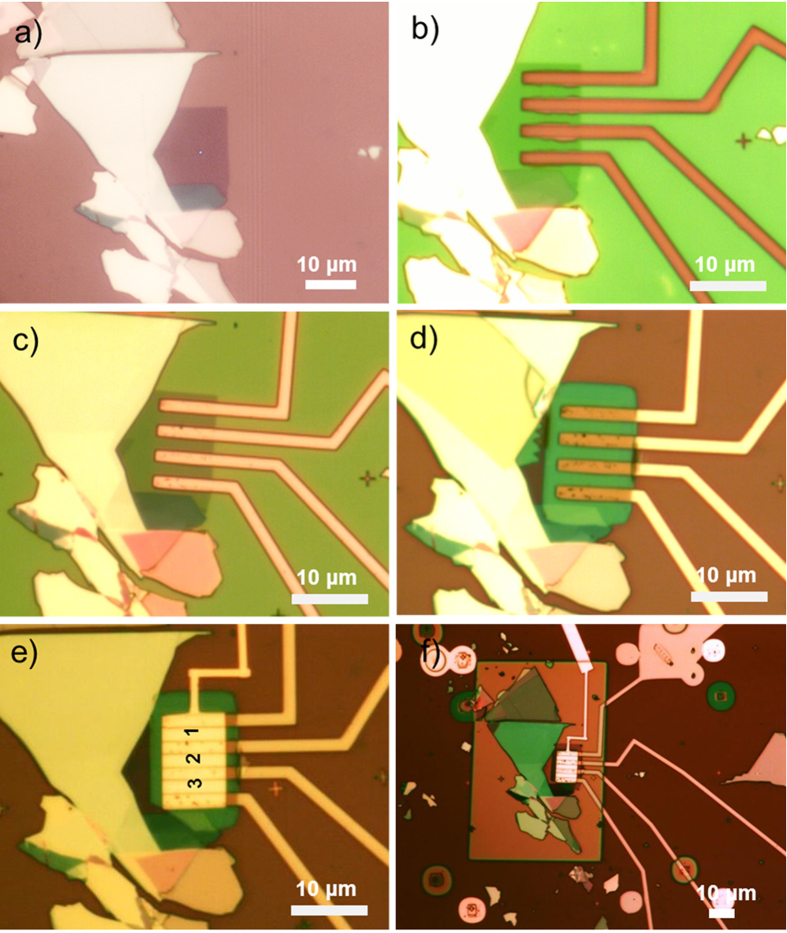
The process flow for making strain-gated FETs (**a**) an optical image of a bi-layer MoS_2_ in faint purple color on a Si/SiO_2_ substrate; (**b**) the material exposed in source drain sites have been etched by helium plasma; (**c**) edge contact metal (Sc/Ni, 50/30 nm) evaporation and lift-off; (**d**) 30 nm HfO_2_ ALD deposition (with 2 nm Al seed) and lift-off, for the bi-layer region only; (**e**) top-gate metal (Ti/Au, 10/60 nm) deposition, the FETs are labeled No. 1, 2 and 3; (**f**) 125 nm SiN_x_ PECVD deposition at 120 °C, capping both the multi-layer and the device area.

Figure 4 shows the optical images captured at different stages of transistor fabrication. Firstly, MoS_2_ flakes were directly exfoliated onto a Si/SiO_2_ substrate and a bi-layer region (faint purple color) was identified (Fig. 4a). The thickness is confirmed by the 22 cm^−1^ difference between the 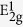 and 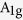 modes and low wavenumber shear mode 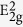 (20 ~25 cm^−1^)^26^ (Fig. S2). After the first e-beam lithography step, the sites for source drain contacts were patterned and etched by helium plasma (Fig. 4b). Instead of using SF_6_^ ^24, helium plasma can prevent potential oxidation of the exposed MoS_2_ atoms. The edge contacts are completed after metal evaporation (Sc/Ni) and lift-off (Fig. 4c). At the completion of the electrical measurement on the back-gate transistors, the bi-layer region is covered by a top-gate dielectric comprised of an AlO_x_/HfO_2_ stack (Fig. 4d). The AlO_x_ layer, which helps to increase the nucleation sites for the atomic layer deposition of HfO_2_, was formed by e-beam evaporation of 2 nm aluminum seeds, followed by its overnight natural oxidation in air^27^. The MoS_2_ transistors were sent for electrical measurement again after Ti/Au were deposited as the top-gate electrode (Fig. 4e). In the last lithography step, the entire MoS_2_ flake, including both the multi-layer and device area, are sealed underneath the PECVD SiN_x_ stress capping layer (Fig. 4f). The recipe was the same as the one used in the Raman characterization.

In total, three FETs are fabricated on this bi-layer MoS_2,_ as labeled on Fig. 4e. To test whether all three are functioning, the output and transfer characteristics were initially taken at the back-gate configuration (Fig. S3). The effective modulation of the drain current by *V*_BG_ tells that all three edge-contacted FETs are working properly, though some non-linearity and discrepancy is still observed in the output plots, which could be due to the difference in contact resistance. In order to make a fair comparison across the three stages of fabrication, from this point on, the discussion is focused on the electrical performance of FET No.2.

**Figure 5:**
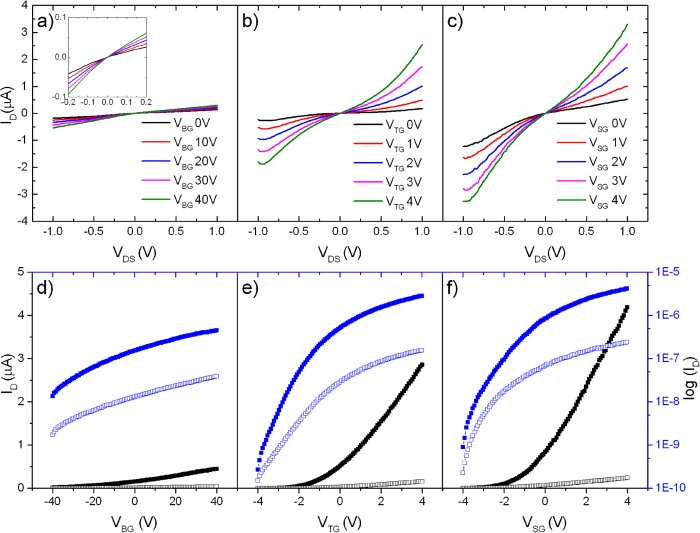
Electrical characterization of FET No. 2. Output plots (**a** to **c**) are in the first row, in which *V*_DS_ is swept from −1 to 1 V. The inset in (**a**) enlarges the area close to the origin. The names of the gate voltages tell which process the plot corresponds to: BG – back-gate, TG – top-gate, SG – strain gate. Gate transfer plots (**d** to **f**) are in the second row, with the linear axis on the left in black color and the log axis on the right in blue. The curves with filled markers correspond to a *V*_DS_ bias of 1 V; those with empty markers have *V*_DS_ of 100 mV.

The full electrical characteristics of FET No. 2, comprising of results from back-gated, top-gated and strain-gated transistor structure, are given in Fig. 5. Output plots are shown in the first row, in which *V*_DS_ is swept from −1 to 1 V. The inset of Fig. 5(a) enlarges the area close to the origin. From −200 to 200 mV, *I*_D_ changes almost linearly with *V*_DS_, indicating an Ohmic-like junction. From Fig. 5(a) to (c), qualitatively, the spreading of the output curves along the vertical axis indicates continuously improved drain current and a more effective current modulation from the top-gated voltages. Of note is that the top-gate voltages were set to 10 times smaller than that for back-gate, for a much thinner top-gate dielectric and the enhanced over 60 times capacitance per unit area. As shown in the following equation, here we use 30 nm thick HfO_2_ with a theoretical relative permittivity of *ε*_*r*_ = 25, and 285 nm thick SiO_2_ with *ε*_*r*_ = 3.9^18^:


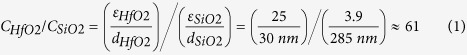


Gate transfer plots are in the second row of Fig. 5. We notice three important results across all three transfer plots: 1) the drain current increases significantly at positive gate voltage, indicating n-type FET; 2) steep slopes of the transfer curves when we switch from back-gate to top-gate; owing to the much improved top-gate capacitance with HfO_2_, the change in slope is later manifested as an evident decrease in sub-threshold swing; 3) the continued increase in drain current from top-gate to strain-gate, which proves that the deposition of the nitride stress liner indeed helps to enhance the transistor’s electrical performance.

**Figure 6:**
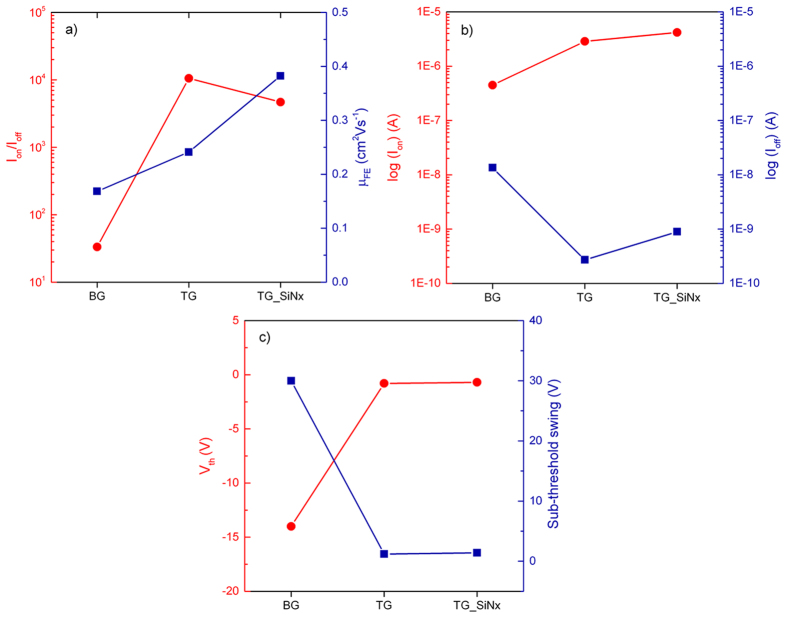
A summary of the carrier transport properties of FET No. 2, encompassing the electrical characterization made at the three stages of device fabrication: back-gate, top-gate and strain-gate.

The extracted carrier transport parameters are plotted in Fig. 6. The “on” and “off” currents are defined as the maximum and the minimum currents in the measured gate voltage range. The mobility values were obtained firstly by curve fitting to each *I*_D_ - *V*_GS_ curve and then calculated by applying the following equation^28^:


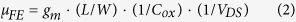


where *C*_ox_ = 1.2 × 10^−8^ F/cm^2^ is the capacitance per unit area of the 285 nm-thick SiO_2_ back- gate dielectric, and *C*_ox_ = 7.4 × 10^−7^ F/cm^2^ for 30 nm HfO_2_ top-gate dielectric. The aspect ratio of transistor No. 2 is W/L = 7.5/1.8 μm. Note that the maximum value of the transconductance 
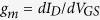
 was used in the calculation. The data plotted in Fig. 6 were all obtained at *V*_DS_ = 1 V. From back- to top-gated FET, quantitatively, we notice a 5 times increase in on-current, ~98% decrease in off-current, significantly improved current on/off ratio from <100 to ~10^4^, and a 43% increase in electron mobility. For the threshold voltage calculated using the linear extrapolation method^29^, the value shrinks from −14 to −0.8 V; so does the sub-threshold swing, drops from 30 to 1.2 V. These improvements are most likely originated from the higher gate capacitance of the more insulative HfO_2_ in the top-gate structure. From top- to strain-gated FET, we find another 58% increase in electron mobility and 46% increase in on-current magnitude, *V*_th_ decreases slightly to −0.7 V, and the sub-threshold swing remains relatively small at 1.4 V. The consistancy in sub-threshold swing indicates that there is no change of the interface quality of the dielectric layer. Distinct piezotronic response of the MoS_2_ was not observed, which is probably because of the opposite orientation of alternating layers in bi-layer 2 H MoS_2_. Flakes with even number of layers are expected to be centrosymmetric and non-piezoelectric^30^. As the nitride PECVD deposition temperature is lower than that for contact annealing, we can conclude that the performance improvement is not due to a better contact, but a direct result from the strain effect induced by the nitride capping layer. Though the magnitude of the mobility is still too low for practical logic device, which is probably a result of the long span of time in multiple e-beam lithograpy steps and repetitive electrical characterizations in between, the ideas presented in this paper can be undoubtedly expanded to other TMDC semiconductor with high intrinsic mobilities. FET No. 3 exhibits similar carrier transport enhancement as FET No. 2, the extracted electron mobilities at the corresponding BG – TG – SG are 0.11, 0.13 and 0.20 cm^2^/Vs, whereas no obvious performance improvement was observed in FET No. 1. It is possible that PECVD gives rough granulated SiN_x_ surface at lower deposition temperature^31^, leading to incoherent stress experienced by the bi-layer MoS_2_ flake.

In summary, we have characterized the strain induction in MoS_2_ through both spectroscopic study and electrical measurement. We have also seen significant improvement in on-current density and mobility, when the FET evolves from back-gated, to top-gated and finally to strain-gated configuration. We conclude that the pioneering approach of strain induction through a nitride stress liner has a benign effect on improving the carrier transport property. It is also an industry-compatible and permanent solution for strain exertion without relying on external substrate bending facilities. Further studies of the strain effect in “all 2D” transistors with perceived greater flexibility would bring in deeper understanding of the tunibility of TMDCs’ electrical properties under mechanical strain.

## Methods

### Bi-layer MoS_2_ sample preparation

The bi-layer MoS_2_ sample for spectroscopic study was initially exfoliated onto a PDMS-based gel-film, and then transferred onto a pre-cleaned oxidized Si substrate through an all-dry micro-manipulation procedure^32^. This additional exfoliation step on gel-film significantly improved the yield of mono- and bi-layer MoS_2_ to almost 100%. The freshly cleaved ultra-thin regions usually come in area of a few μm^2^, feasible for multiple transistor fabrication. The second bi-layer MoS_2_ sample used in strain-gated transistor fabrication was prepared following the conventional Scotch-tape based mechanical exfoliation using bulk crystal. Raman and photoluminescence spectra were acquired under ambient conditions with a Horiba LabRAM HR spectrometer equipped with a 523 nm laser supply and an 1800 lines/mm grating. A 100 × objective was used for focusing the laser to an approximately 1 μm spot onto the sample. The laser power is < 1 mW to prevent sample heating. Thin film thickness measurement was carried out under tapping mode from the AFM module of the spectrometer.

### Strained bi-layer MoS_2_ band structure calculation

The band dispersions and band gap energies of bilayer MoS_2_ with and without strains were calculated using the Vienna ab initio simulation package (VASP)^33^,^34^,^35^ in the projected-augmented-wave method^36^. The generalized gradient approximation (GGA) of the Perdew-Burke-Ernzerhof form^37^,^38^,^39^ is used for the exchange correlation energy. The vdW interactions between the two MoS_2_ layers are accounted by using the DFT-D2 method of Grimme^40^. The kinetic energy cutoff for our calculation is set as 500 eV. The lattice constant of bilayer MoS_2_ without strain is about 3.179 Å. For all structural relaxations, the convergence tolerance on the Hellmann-Feynman forces is less than 0.01 eV/Å. An 8 × 8 × 1 Monkhorst-Pack k-point mesh is used for 2D films. The vacuum layer added to the system is nearly 20 Å. The other strained systems are all calculated under the same setting.

### Edge contacts and MoS_2_ transistors fabrication

A degenerately boron-doped (0.001~0.005 Ω•cm) silicon substrate with 285 nm SiO_2_ capping layer, served as the global back gate and gate dielectric; silicon nitride PECVD deposition: Plasma-therm Unaxis 790, deposition pressure 900 mTorr, power 25 W, RF frequency 13.56 MHz, 2% SiN_4_ 200 sccm, NH_3_ 4 sccm, N_2_ 900 sccm, deposition time 10 mins at 120 °C, film thickness 125 nm; helium plasma for opening edge contacts: STS Reactive Ion Etcher Dielectric System, helium flow rate 50 sccm, 75 W, 20 s; HfO_2_ ALD deposition: Cambridge Nanotech Savannah 100, 250 cycles at 120 °C, film thickness 30 nm; Metal evaporation: Temescal BJD 1800 system, aluminum seed (2 nm, 0.1 Å/s), source drain electrodes (Sc/Ni, 50/30 nm), back-gate electrode (Ti/Au, 10/100 nm), top-gate electrode (Ti/Au, 10/60 nm); post-HfO_2_ deposition contacts annealing: RTP-600S system, 200 °C, Ar, 1 hour. The FETs were characterized in a shielded probe station connected to an Agilent 4155 C semiconductor parameter analyzer. The entire measurement was carried out at room temperature in air.

## Additional Information

**How to cite this article:** Chai, Y. *et al*. Strain Gated Bilayer Molybdenum Disulfide Field Effect Transistor with Edge Contacts. *Sci. Rep.* **7**, 41593; doi: 10.1038/srep41593 (2017).

**Publisher's note:** Springer Nature remains neutral with regard to jurisdictional claims in published maps and institutional affiliations.

## Supplementary Material

Supplementary Information

## References

[b1] Thompson S. E. . A 90-nm Logic Technology Featuring Strained-Silicon. IEEE Transactions on Electron Devices 51, 1790–1797 (2004).

[b2] Xiong W. W. In FinFETs and Other Multi-Gate Transistors 49–111 (Springer, 2008).

[b3] Thompson S. E. . A 90 nm Logic Technology Featuring 50 nm Strained Silicon Channel Transistors, 7 layers of Cu Interconnects, Low k ILD, and 1 μm^2^ SRAM Cell. IEDM Technical Digest. 61–64 (IEEE, 2002).

[b4] Ieong M. K. . Silicon Device Scaling to the Sub-10-nm Regime. Science 306, 2057–2060 (2004). 15604400 10.1126/science.1100731

[b5] Ito S. . Mechanical stress effect of etch-stop nitride and its impact on deep submicron transistor design. In Electron Devices Meeting, 2000. IEDM’00. Technical Digest. International 247–250 (IEEE, 2000).

[b6] Ge C.-H. . Process-strained Si (PSS) CMOS technology featuring 3D strain engineering. In Electron Devices Meeting, 2003. IEDM’03 Technical Digest. IEEE International 3–7 (IEEE, 2003).

[b7] Ghani T. . A 90 nm high volume manufacturing logic technology featuring novel 45 nm gate length strained silicon CMOS transistors. In Electron Devices Meeting, 2003. IEDM’03 Technical Digest. IEEE International 11–6 (IEEE, 2003).

[b8] Mistry K. . Delaying forever: Uniaxial strained silicon transistors in a 90nm CMOS technology. in Symposium on VLSI Technology 50–51 (2004).

[b9] Van der Zande A. & Hone J. Inspired by Strain. Nature Photonics 6, 804–806 (2012).

[b10] Bertolazzi S., Brivio J. & Kis A. Stretching and Breaking of Ultrathin MoS_2_. ACS Nano 5, 9703–9709 (2011). 22087740 10.1021/nn203879f

[b11] Yun W. S., Han S. W., Hong S. C., Kim I. G. & Lee J. D. Thickness and strain effects on electronic structures of transition metal dichalcogenides: 2H-MX_2_ semiconductors (M = Mo, W; X = S, Se, Te). Physical Review B 85, 033305 (2012).

[b12] Zeng L., Xin Z., Chang P. & Liu X. Strain effects on monolayer MoS_2_ field effect transistors. Japanese Journal of Applied Physics 54, 04DC17 (2015).

[b13] Yang L. . Lattice strain effects on the optical properties of MoS_2_ nanosheets. Scientific Reports 4 (2014). 10.1038/srep05649 PMC4090623 25008782

[b14] Conley H. J. . Bandgap Engineering of Strained Monolayer and Bilayer MoS_2_. Nano Letters 13, 3626–3630 (2013). 23819588 10.1021/nl4014748

[b15] Zhu C. R. . Strain tuning of optical emission energy and polarization in monolayer and bilayer MoS_2_. Physical Review B 88, 121301 (2013).

[b16] He K., Poole C., Mak K. F. & Shan J. Experimental Demonstration of Continuous Electronic Structure Tuning via Strain in Atomically Thin MoS_2_. Nano Letters 13, 2931–2936 (2013). 23675872 10.1021/nl4013166

[b17] Hui Y. Y. . Exceptional Tunability of Band Energy in a Compressively Strained Trilayer MoS_2_ Sheet. ACS Nano 7, 7126–7131 (2013). 23844893 10.1021/nn4024834

[b18] Tsai M.-Y. . Flexible MoS_2_ Field-Effect Transistors for Gate-Tunable Piezoresistive Strain Sensors. ACS Applied Materials & Interfaces 7, 12850–12855 (2015). 26010011 10.1021/acsami.5b02336

[b19] Gatzen H. H., Saile V. & Leuthold J. In Micro and Nano Fabrication: Tool and Processes, 65–203 (Springer Berlin Heidelberg, 2015).

[b20] Mackenzie K. D., Johnson D. J., DeVre M. W., Westerman R. J. & Reelfs B. H. Stress control of Si-based PECVD dielectrics. In Electrochemical Society Meeting 148–159, Pennington, NJ (2005).

[b21] Splendiani A. . Emerging Photoluminescence in Monolayer MoS_2_. Nano Letters 10, 1271–1275 (2010). 20229981 10.1021/nl903868w

[b22] Lee C., Yan H., Brus L. E., Heinz T. F., Hone J. & Ryu S. Anomalous Lattice Vibrations of Single and Few-Layer MoS_2_. ACS Nano 4, 2695–2700 (2010). 20392077 10.1021/nn1003937

[b23] Plechinger G. . Low-temperature photoluminescence of oxide-covered single-layer MoS2. Physica Status Solidi (RRL) - Rapid Research Letters 6, 126–128 (2012).

[b24] Chai Y. . Making one-dimensional electrical contacts to molybdenum disulfide-based heterostructures through plasma etching. Physica Status Solidi A. (2016).

[b25] McDonnell S., Addou R., Buie C., Wallace R. M. & Hinkle C. L. Defect-Dominated Doping and Contact Resistance in MoS_2_. ACS Nano 8, 2880–2888 (2014). 24484444 10.1021/nn500044q

[b26] Zeng H. . Low-frequency Raman modes and electronic excitations in atomically thin MoS 2 films. Physical Review B 86, 241301 (2012).

[b27] Zhu W. . Electronic transport and device prospects of monolayer molybdenum disulphide grown by chemical vapour deposition. Nature Communications 5 (2014). 10.1038/ncomms4087 24435154

[b28] Sze S. M. & Ng K. K. In Physics of Semiconductor Devices (3rd ed) chap. 6 (Wiley-Interscience, 2007).

[b29] Schroder D. K. In Semiconductor Material and Device Characterization, p.223. (John Wiley & Sons, Inc., 2006).

[b30] Wu W. . Piezoelectricity of single-atomic-layer MoS_2_ for energy conversion and piezotronics. Nature 514, 470–474 (2014). 25317560 10.1038/nature13792

[b31] Semendy F. . Characterization of Multi Temperature and Multi RF Chuck Power Grown Silicon Nitride Films by PECVD and ICP Vapor Deposition. Army Resarch Laboratory, ARL-TR-5105 (2010).

[b32] Castellanos-Gomez A. . Deterministic transfer of two-dimensional materials by all-dry viscoelastic stamping. 2D Materials 1, 011002 (2014).

[b33] Kresse G. & Furthmüller J. Efficient iterative schemes for ab initio total-energy calculations using a plane-wave basis set. Physical Review B 54, 11169 (1996). 10.1103/physrevb.54.11169 9984901

[b34] Kresse G. & Hafner J. Ab initio molecular dynamics for liquid metals. Physical Review B 47, 558 (1993). 10.1103/physrevb.47.558 10004490

[b35] Kresse G. & Furthmüller J. Efficiency of ab-initio total energy calculations for metals and semiconductors using a plane-wave basis set. Computational Materials Science 6, 15 (1996). 10.1103/physrevb.54.11169 9984901

[b36] Blöchl P. E. Projector augmented-wave method. Physical Review B 50, 17953 (1994). 10.1103/physrevb.50.17953 9976227

[b37] Perdew J. P. . Atoms, molecules, solids, and surfaces: Applications of the generalized gradient approximation for exchange and correlation. Physical Review B 46, 6671 (1992). 10.1103/physrevb.46.6671 10002368

[b38] Wang Y. & Perdew J. P. Correlation hole of the spin-polarized electron gas, with exact small-wave-vector and high-density scaling. Physical Review B 44, 13298 (1991). 10.1103/physrevb.44.13298 9999531

[b39] Kresse G. & Joubert D. From ultrasoft pseudopotentials to the projector augmented-wave method. Physical Review B 59, 1758 (1999).

[b40] Harl J., Schimka L. & Kresse G. Assessing the quality of the random phase approximation for lattice constants and atomization energies of solids. Physical Review B 81, 115126 (2010).

