# Supplementary material for: Strain Gated Bilayer Molybdenum Disulfide Field Effect Transistor with Edge Contacts
Source: Sci Rep. 2017 Feb 10;7:41593. doi: 10.1038/srep41593 (PMC5301248; doi:10.1038/srep41593)
Supplement: Supplementary Information [file srep41593-s1.doc]

**Supplementary Information**

**Strain Gated Bilayer Molybdenum Disulfide Field Effect Transistor with Edge Contacts**

Yu Chai^1^, Shanshan Su^2^, Dong Yan^3^, Mihrimah Ozkan^2^, Roger Lake^2^ and Cengiz S. Ozkan^1,3*^

^1^Materials Science and Engineering Program, University of California, Riverside, CA 92521 USA

^2^Department of Electrical and Comp. Engineering, University of California, Riverside, CA 92521 USA

^3^Department of Mechanical Engineering, University of California, Riverside, CA 92521 USA
^4^Center for Nanoscale Science & Engineering, University of California, Riverside, CA 92521 USA

Correspondence and requests for materials should be addressed to

C. S. O. (cozkan@engr.ucr.edu)


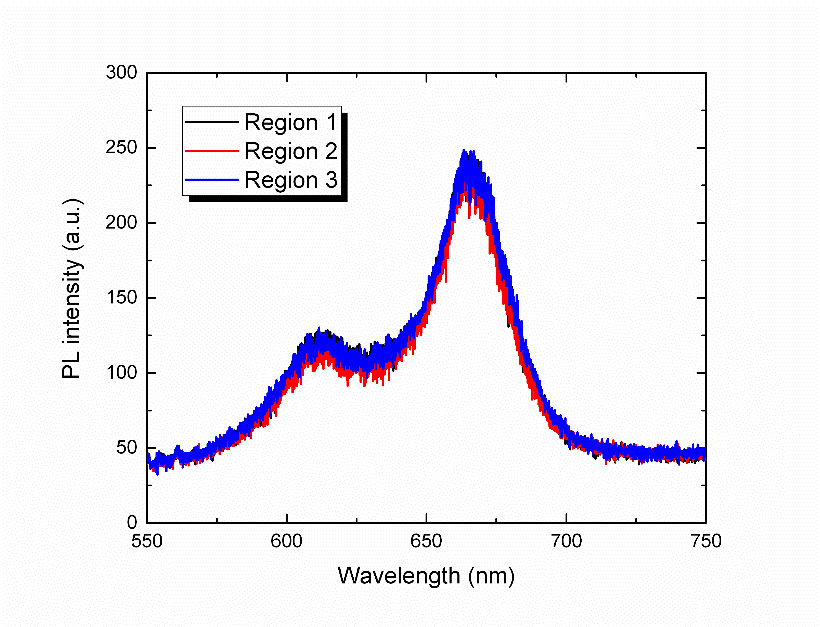

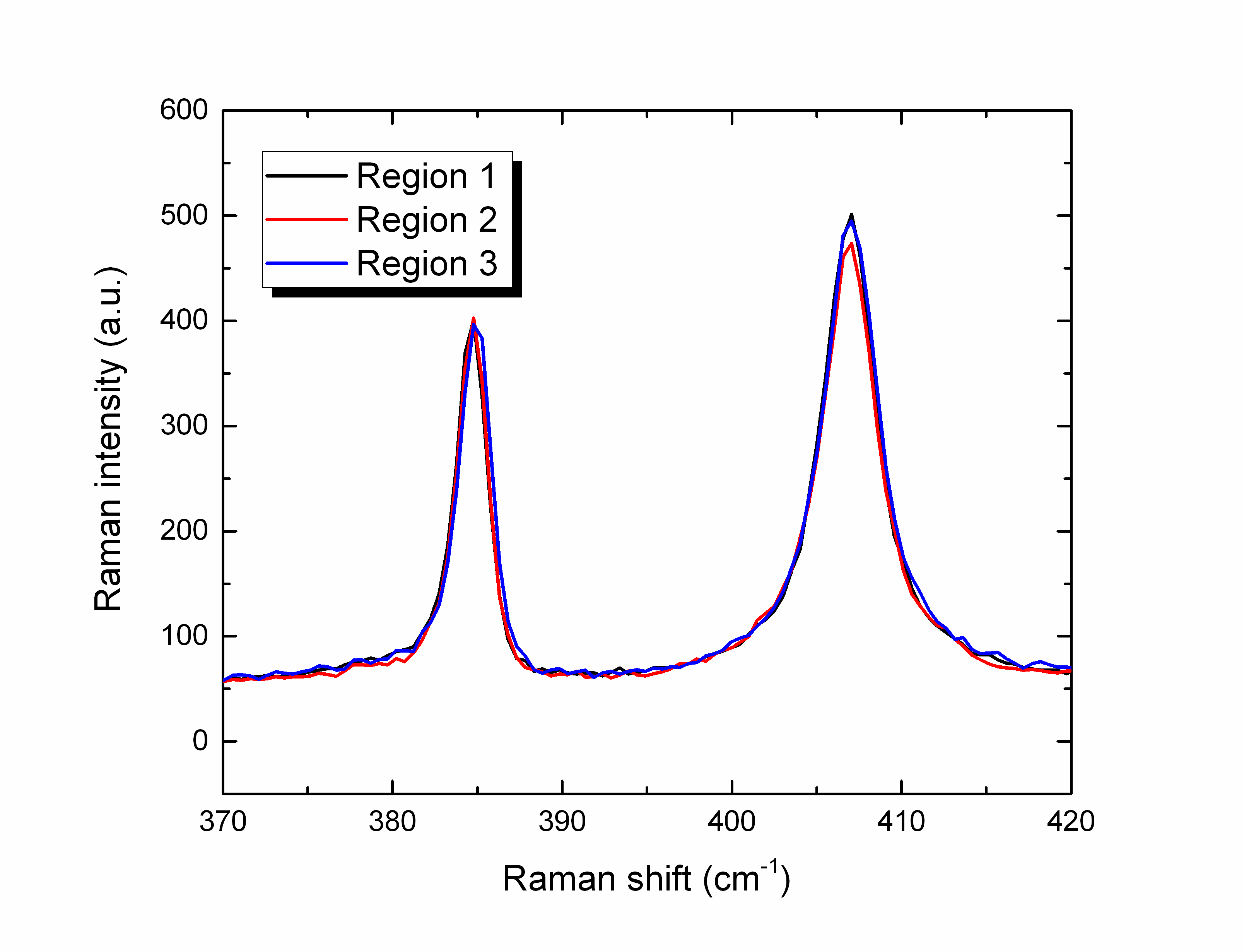


Figure S1. A test on the homogeneity of the bi-layer MoS_2_ sample shown in Fig.1. The PL and Raman spectra taken from the as-prepared sample are re-plotted in overlapping format to show the similarities in peak intensity among the three regions.


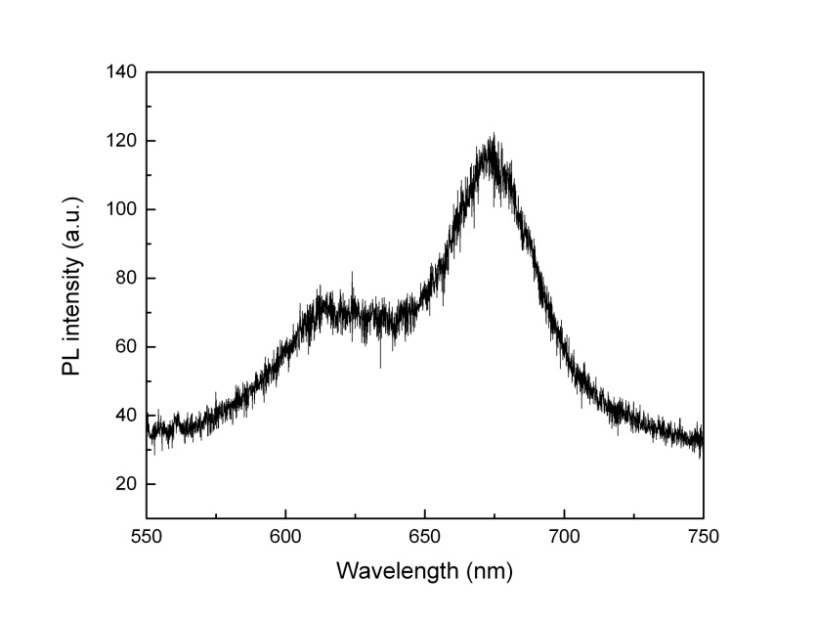

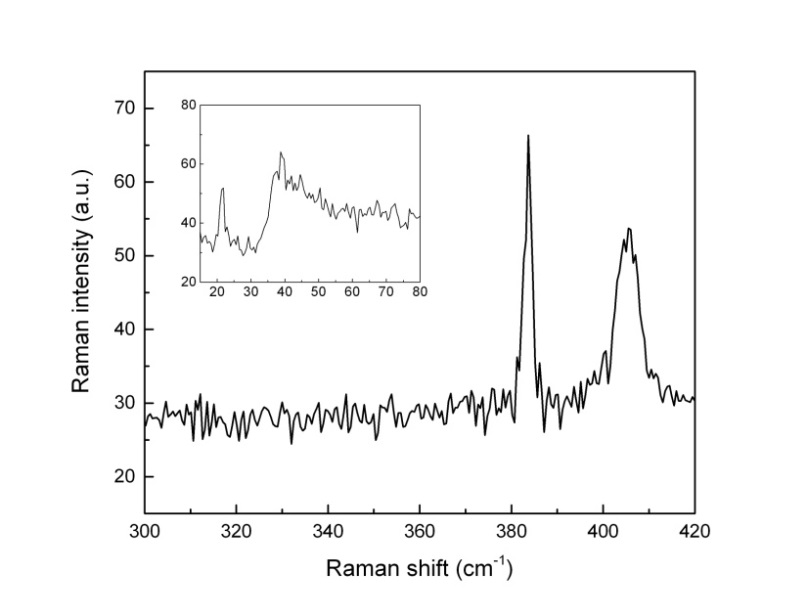


Figure S2. The Raman and PL spectra of the bi-layer MoS_2_ sample selected for device fabrication. The bi-layer thickness is confirmed by the 22 cm^-1^ difference between the
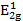
 and
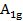
 modes and a low wavenumber shear mode
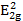
 (20 ~ 25 cm^-1^) ^26^.

**
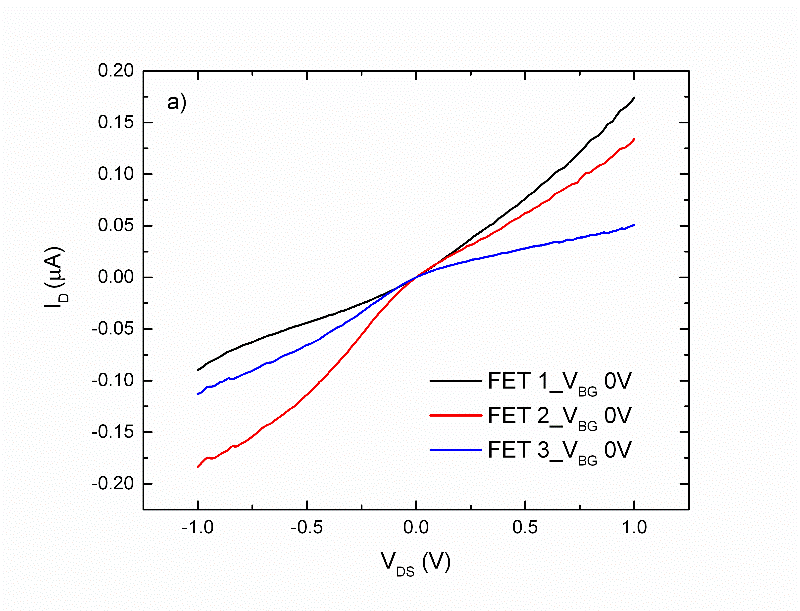

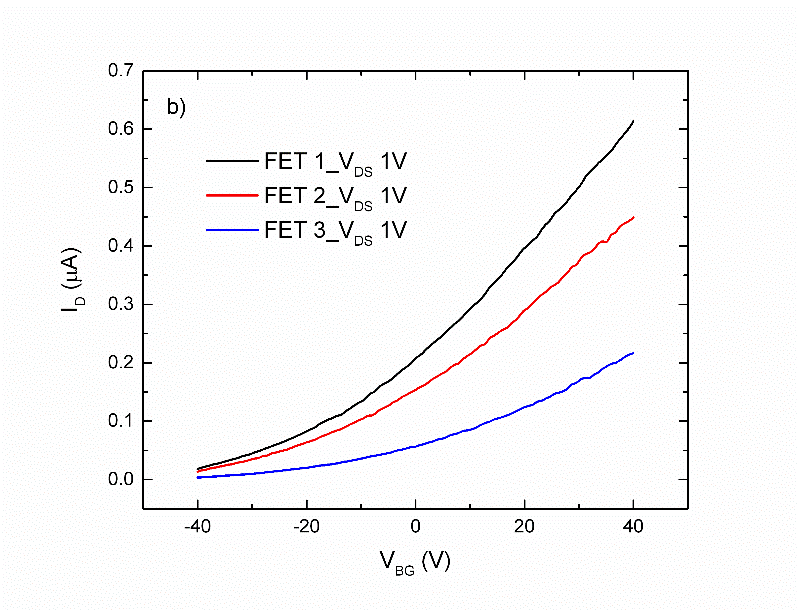
**

Figure S3. I-V repeatability test on the three transistors labeled on Fig. 4e, at the point that the back-gate configuration was completed. (a) in the output plot, *V*_BG_ is set to zero, and *V*_DS_ is swept from -1 to 1V; (b) in the complementary transfer plot, *V*_DS_ is fixed at 1V, while the *V*_BG_ is swept from -40 to 40V. The effective modulation of the drain current by *V*_BG_ tells that all three edge-contacted FETs are working properly, though some non-linearity and discrepancy is still observed in the output plots, which could be due to the difference in contact resistance.
